# Performance of Lead-Free versus Lead-Based Hunting Ammunition in Ballistic Soap

**DOI:** 10.1371/journal.pone.0102015

**Published:** 2014-07-16

**Authors:** Felix Gremse, Oliver Krone, Mirko Thamm, Fabian Kiessling, René Hany Tolba, Siegfried Rieger, Carl Gremse

**Affiliations:** 1 Experimental Molecular Imaging, University Hospital, RWTH Aachen University, Aachen, Germany; 2 Leibniz Institute for Zoo and Wildlife Research, Berlin, Germany; 3 Laboratory Animal Science, University Hospital, RWTH Aachen University, Aachen, Germany; 4 Wildlife Biology, Management and Hunting Practice, HNE Eberswalde, Eberswalde, Germany; USGS National Wildlife Health Center, United States of America

## Abstract

**Background:**

Lead-free hunting bullets are an alternative to lead-containing bullets which cause health risks for humans and endangered scavenging raptors through lead ingestion. However, doubts concerning the effectiveness of lead-free hunting bullets hinder the wide-spread acceptance in the hunting and wildlife management community.

**Methods:**

We performed terminal ballistic experiments under standardized conditions with ballistic soap as surrogate for game animal tissue to characterize dimensionally stable, partially fragmenting, and deforming lead-free bullets and one commonly used lead-containing bullet. The permanent cavities created in soap blocks are used as a measure for the potential wound damage. The soap blocks were imaged using computed tomography to assess the volume and shape of the cavity and the number of fragments. Shots were performed at different impact speeds, covering a realistic shooting range. Using 3D image segmentation, cavity volume, metal fragment count, deflection angle, and depth of maximum damage were determined. Shots were repeated to investigate the reproducibility of ballistic soap experiments.

**Results:**

All bullets showed an increasing cavity volume with increasing deposited energy. The dimensionally stable and fragmenting lead-free bullets achieved a constant conversion ratio while the deforming copper and lead-containing bullets showed a ratio, which increases linearly with the total deposited energy. The lead-containing bullet created hundreds of fragments and significantly more fragments than the lead-free bullets. The deflection angle was significantly higher for the dimensionally stable bullet due to its tumbling behavior and was similarly low for the other bullets. The deforming bullets achieved higher reproducibility than the fragmenting and dimensionally stable bullets.

**Conclusion:**

The deforming lead-free bullet closely resembled the deforming lead-containing bullet in terms of energy conversion, deflection angle, cavity shape, and reproducibility, showing that similar terminal ballistic behavior can be achieved. Furthermore, the volumetric image processing allowed superior analysis compared to methods that involve cutting of the soap blocks.

## Introduction

### Lead-related Problems

The toxicity of lead for humans is well-studied [1] and has been associated with myocardial infarction and stroke mortality [2], decreased brain volume [3], and even elevated crime levels [4]. There is no known lower threshold below which the lead concentration is considered harmless [1], [5]. Furthermore, lead is not required by the human body like other essential trace elements. Children are especially vulnerable to lead poisoning, which results in intellectual impairment [6]–[8] and reduced growth [9]. The removal of lead from gasoline and house paint has greatly reduced the overall lead intake [1], and the remaining sources of lead intoxication are receiving more attention.

In typical semi-jacketed hunting bullets, lead constitutes the main part of the projectile mass [10]. Upon impact, these bullets expand from their aerodynamic shape and increase their cross-sectional area to increase the energy release in the target. The extreme forces acting on the bullets result in strong fragmentation, creating hundreds of small lead fragments [11]–[13]. Due to the high amount and small size of the fragments, a complete removal is not possible as experiments and radiographic investigation of meat packages have shown [14], [15]. Ingested lead is dissolved by stomach acids [16], enters the blood circulation, is distributed among internal organs, concentrates in liver and kidneys, and is stored in bones as long-term deposit [1]. Elevated lead intake is related to the intensity of meat consumption and particularly evident for subsistence hunters [7], [17]–[19].

Lead ammunition also poses a problem for large birds of prey, which scavenge on the lead containing entrails left by hunters [20]. Many studies have identified ammunition-based lead as the principal source for the heavy lead poisoning in the endangered Californian condor [21], [22]. Similar problems affect bald eagles [23], golden eagles [24], Turkey Vultures [25], and White-tailed Sea-eagles [10]. A partial ban of lead ammunition in California in 2008 resulted in a strong reduction of lead poisoning [24]. While the insight into effects on scavenging birds is relatively young, the poisoning of waterfowl through ingestion of lead-based shotgun pellets is well-known and resulted in much earlier legal restrictions [26].

### Lead-free Alternatives

Lead-free hunting bullets are typically composed of copper or brass (an alloy of copper and zinc) instead of lead. Due to the lower density these bullets are often longer or lighter, and in the latter case need to be faster to transport the same amount of kinetic energy [27]. Lead-free bullets retain most of the mass and produce no or few fragments [11], [28] which pose no known health-risk to humans [29]–[31] since copper and zinc are essential trace elements for humans and are tolerated in low amounts [32]. Since these materials are harder than lead, the bullets need to be manufactured differently to expand and release the energy within the target, e.g. by a drilled hole in the tip [27]. Furthermore, adjustments in bullet design are required to avoid damaging of the barrel due to the harder material [33].

In a recent review [34], price, availability, and effectiveness of lead-free ammunition was assessed and found to be equally good as lead ammunition. It was constituted however, that: “There has been little formal research published on the effectiveness of lead-free rifle bullets, and this may contribute to the reluctance of some hunters to embrace lead-free ammunition”. Indeed, only few studies have investigated the accuracy and killing performance of lead-free ammunition [35]–[39]. Concerns were raised by practitioners regarding lead-free bullet performance and selection [27], [39], [40] which can be attributed to the absence of thorough investigations of the terminal ballistic behavior [41]. However, given the “overwhelming scientific evidence on the toxic effects of lead on human and wildlife health” [42], several authors, including a consortium of 30 scientists, recommended to reduce and eventually eliminate the use of lead-containing hunting ammunition world-wide [34], [42]–[44].

### Terminal Ballistic Experiments

The terminal ballistic properties of a bullet can be analyzed by shooting in ballistic soap or gelatin blocks [45]. Gelatin is elastic and does not maintain the temporary cavity created by the bullet but it can be imaged with high speed cameras [45]. In contrast to gelatin, soap blocks have the advantage to maintain the cavity created by the bullet while showing otherwise similar characteristics [45]. Ballistic soap is generally dull, requiring cutting or non-destructive imaging methods to assess the shape of the cavity [46]. For gelatin the total crack length, i.e. the amount of fissures, can be assessed as an estimate of the wound damage by cutting or non-destructive imaging [47], [48].

Experiments with soap or gelatin may not correspond to real tissue exactly. However, they are an accepted surrogate for animal tissue and are useful to analyze in vitro the bullet in a simplified medium under reproducible conditions [45], [49]. This is especially important also for animal welfare because otherwise the bullets would get used for hunting wild animals without proper testing. For these experiments, an important free parameter is the speed of the bullet, corresponding to the shooting distance, which may range between a few and several hundred meters in hunting scenarios. To thoroughly characterize a bullet, shots at different impact speeds are therefore required.

Computed tomography (CT) allows non-destructive tomographic imaging of the soap or gelatin blocks. CT was used to visualize the cavity and the metal fragments in 3D and to assess the cross-sectional area of the cavity by manual measurements at multiple slices [46]. This technique was used to investigate the effect of barrel shortening [50] and to assess the cavity shape of spherical bullets used in muzzle-loading weapons [51]. CT was used to show that the majority of venison packages [14] is contaminated by lead fragments. CT has even been used to compare wound channels of lead-containing and lead-free hunting ammunition in wild undulates, where 34 dead wild ungulates were analyzed by CT and no differences were found between lead-containing and lead-free ammunition [38]. To the best of our knowledge, lead-containing and lead-free bullets have not been systematically compared using soap block experiments and CT. We selected three lead-free bullets (Table 1) to cover three different types (dimensionally stable, partially fragmenting and deforming), according to previous classifications [38]. Some of the lead-free bullets and the lead-containing bullet were used in previous studies [10], [34], [36], [38], [45] and were among the most favored bullets in a recent field study in Germany where more than 11, 000 field records were analyzed [37].

**Table 1:** Bullet types.

Name	Abbreviation	Materials	Type	Caliber[mm]	Weight[g]	Weight[gr]
Impala LS	ILS	Brass	Dimensionally stable	7.62	8.45	130.40
Brennecke TAG	TAG	Copper	Fragmenting	7.62	10.07	155.40
Barnes TSX	TSX	Copper	Deforming	7.62	10.71	165.28
Norma Vulcan	NVU	Lead/Copper	Deforming	7.62	11.68	180.25

Four commercially available bullets were used for the experiments.

### Aim of the Study

The aim of our study is to utilize and show the value of computed tomography and volumetric image processing to assess and compare the terminal ballistic behavior of lead-based and lead-free hunting bullets in ballistic soap blocks. Our questions are: What are the differences in the energy-to-volume conversion between the bullets at different speeds? Does the fragmentation differ between the bullets tested here? Do the bullets display specific cavity shapes?

## Materials and Methods

### Bullet Types

Four different bullet types with 7.62 mm diameter were used (Table 1). The Impala LS (ILS) bullet is a dimensionally stable, i.e. non-deforming, light high-speed brass bullet. The cone-shaped tip causes the bullet to tumble inside the target, thereby increasing the cross-sectional diameter to release the energy. Both copper bullets, Brennecke TAG (TAG) and Barnes TSX (TSX), have an aerodynamic shape and are designed to expand inside the target. The TAG bullet has an aluminum tip to support the expansion of the bullet, while the TSX bullet has a drilled hole in the tip for that purpose. The Norma Vulcan (NVU) bullet is a commonly used semi-jacketed bullet with a copper alloy jacket and a lead filling [10].

### Ballistic Soap Experiments

The ballistic experiments were performed at the DEVA (Deutsche Versuchs- und Prüfanstalt für Jagd- und Sportwaffen e. V., Altenbeken, Germany) by using glycerin soap blocks (Enzian Seifen GmbH & Co. KG, Metzingen, Germany) of size 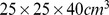 positioned 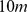 in front of a test machine barrel. Soap blocks where tested for appropriate consistency with an air gun which should result in a penetration depth of 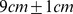 when using caliber 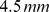 at 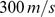 [45]. Powder loading was adjusted by an expert to approximately reach the desired impact speeds. The mounted rifle was set up to hit the center of the soap block. The speed of the bullet was measured by light barriers (LS-1200, Kurzzeitmesstechnik Werner Mehl, Germany) upon entering and exiting the soap block. The exiting bullet fragments were caught in cotton and weighed. From the speed measurements and the measured weights of the initial and exiting masses the deposited energy was calculated.

For each bullet type, four different impact speeds were tested with one repetition resulting in 32 blocks in total. The speeds for the light and fast brass bullet (ILS) were 600, 700, 800, 900 m/s. For the slightly heavier TAG and TSX bullets, speeds of 550, 650, 750, 850 m/s were used. For the heaviest bullet, the lead-containing NVU bullet, 500, 600, 700 and 800 m/s were used. These intervals were chosen to cover the range of bullet speeds that can be expected from typical settings in the field [37].

**Figure 1:**
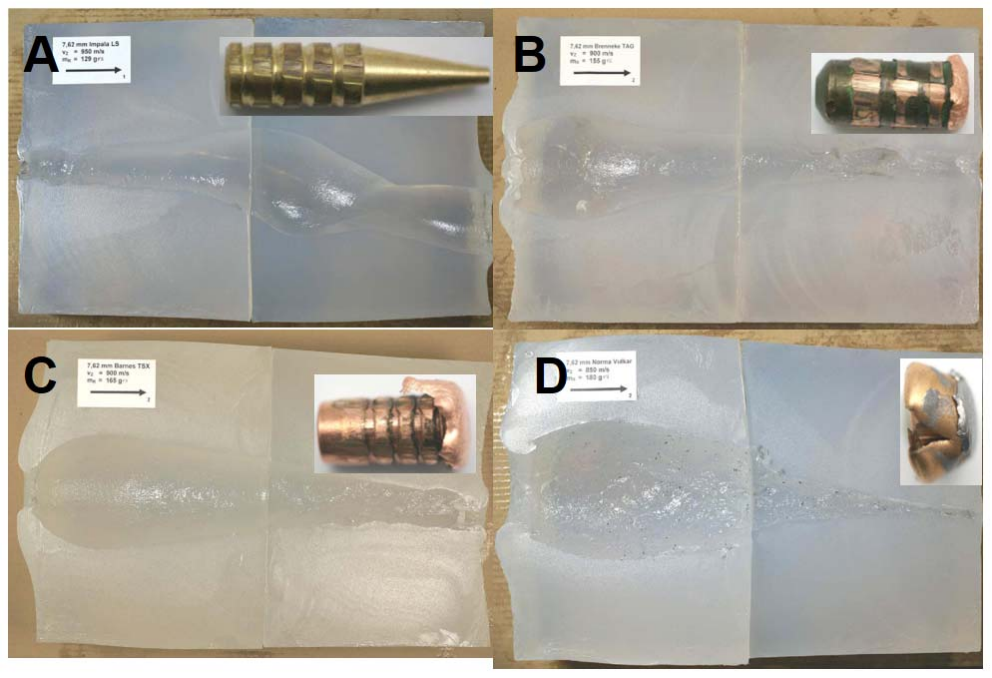
Cut soap blocks for different bullet types. Exiting bullet fragments are shown as inlay. (A) Lead-free Impala LS (ILS). (B) Lead-free Brennecke TAG (TAG). (C) Lead-free Barnes TSX (TSX). (D) Lead-based Norma Vulkan (NVU).

After shooting, the soap blocks were immediately transported to a CT and scanned. Subsequently, the blocks were cut into 4 parts using a wire (Fig. 1). The open block was imaged using a digital camera, which was positioned at a fixed distance from the block. In the photograph, the cavity was manually delineated using mouse clicks at arbitrary distances averaging one measurement per 2 cm and these measurements were used to compute a model of the cavity by assuming segment-wise truncated cones [45]. Based on this model, the total volume was computed and the cross-sectional area was determined as function of the penetration depth.

### CT Scanning

The soap blocks were scanned using a clinical 64-channel CT (Brilliance 64, Philips, Hamburg, Germany), operating at voltage 120 kV, power 192 mA, and exposure time 781 ms. The blocks were positioned to be aligned with the main axis of the CT. Reconstructions at voxel size 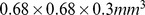 were performed using a medium sharp kernel.

### 3D Image Analysis

**Figure 2:**
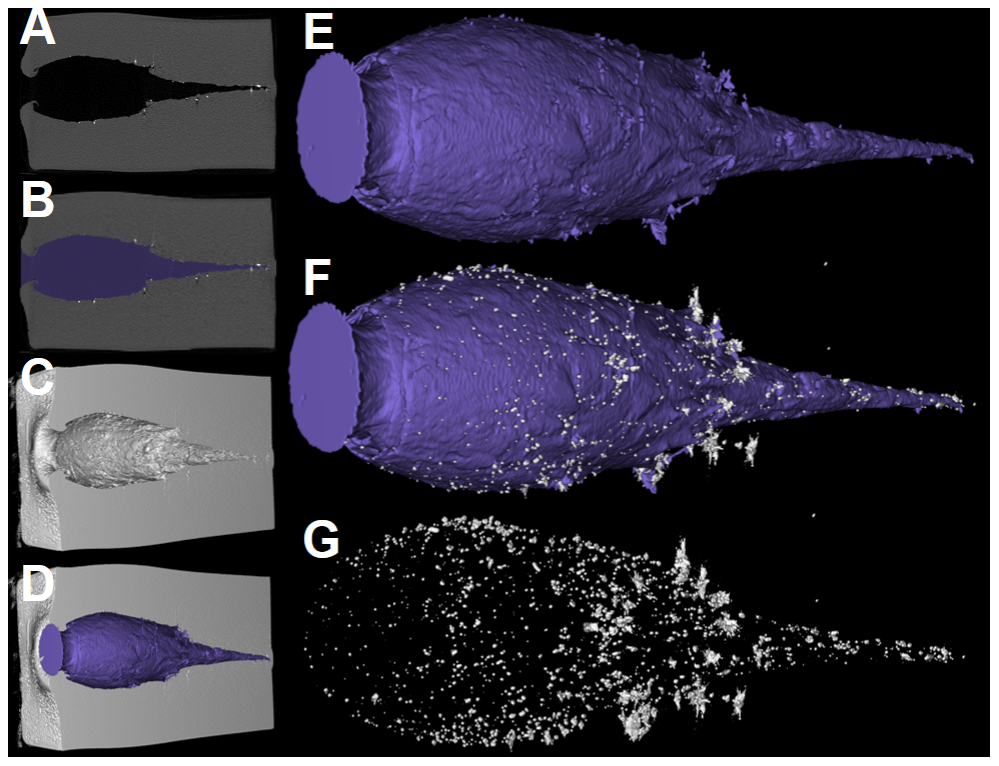
CT method. (A) CT slice of the soap block with a cavity. Air outside the block and in the cavity appears black. The soap appears gray and the metal fragments as small white spots. (B) Segmented cavity shown in blue. (C, D) 3D renderings of the virtually cut soap block and the segmented cavity. (E) 3D rendering of the cavity. (F) Cavity with lead fragments. (G) Cloud of lead fragments.

Based on a fixed threshold (50% of the soap intensity) the soap block was segmented using region growing. The first and last slices of the cavity were manually selected and the cavity was segmented using region growing. The segmentation was represented as a binary mask which allows a 3D visualization of the cavity (Fig. 2) and enables computation of the volume and other parameters [52]. Using a higher threshold (2000 Hounsfield units) well above the soap intensity, the metal fragments were segmented and automatically counted. Small metal fragments which were close to large fragments were not counted since these were likely to be caused by streaking artifacts. By principal component analysis, the best approximating axis directions were determined for the cavity and the soap block, and the deflection angle was set as the angle between these axis directions. Based on the cavity segmentation, a distance map [53] was computed, which holds the distance between the voxel and the closest point on the cavity. The maximum value and position of this distance map indicates the radius and position of the maximal fitting sphere [53]. Its z-position was used to quantify the depth of maximal damage. The whole processing was performed using the Imalytics Preclinical Software (developed at ExMI in cooperation with Philips), which allows interactive segmentation and visualization of volumetric data sets.

### Statistics

Linear regression analysis was performed to find dependencies between variables, such as deposited energy and the number of metal fragments. Slopes of regression lines where compared using analysis of covariance (ANCOVA). Angular stability and depth of maximum damage were compared between bullet types using a one-way analysis of variance and Tukey's multiple comparison test. Regression fits were computed using Microsoft Excel 2007, and statistical analysis for significance was performed using GraphPad Prism 5. Regression fits were compared using the Akaike information criterion which takes the number of parameters into account. The coefficient of determination is denoted as 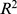. Linear and quadratic fits modeling the volume over the energy were computed with the constraint that the curves go through the origin since this is a reasonable assumption and reduces the degree of freedom by one. A level of 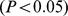 was assumed to be significant. Values are reported as 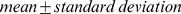.

## Results

### Energy to Volume Conversion

**Figure 3:**
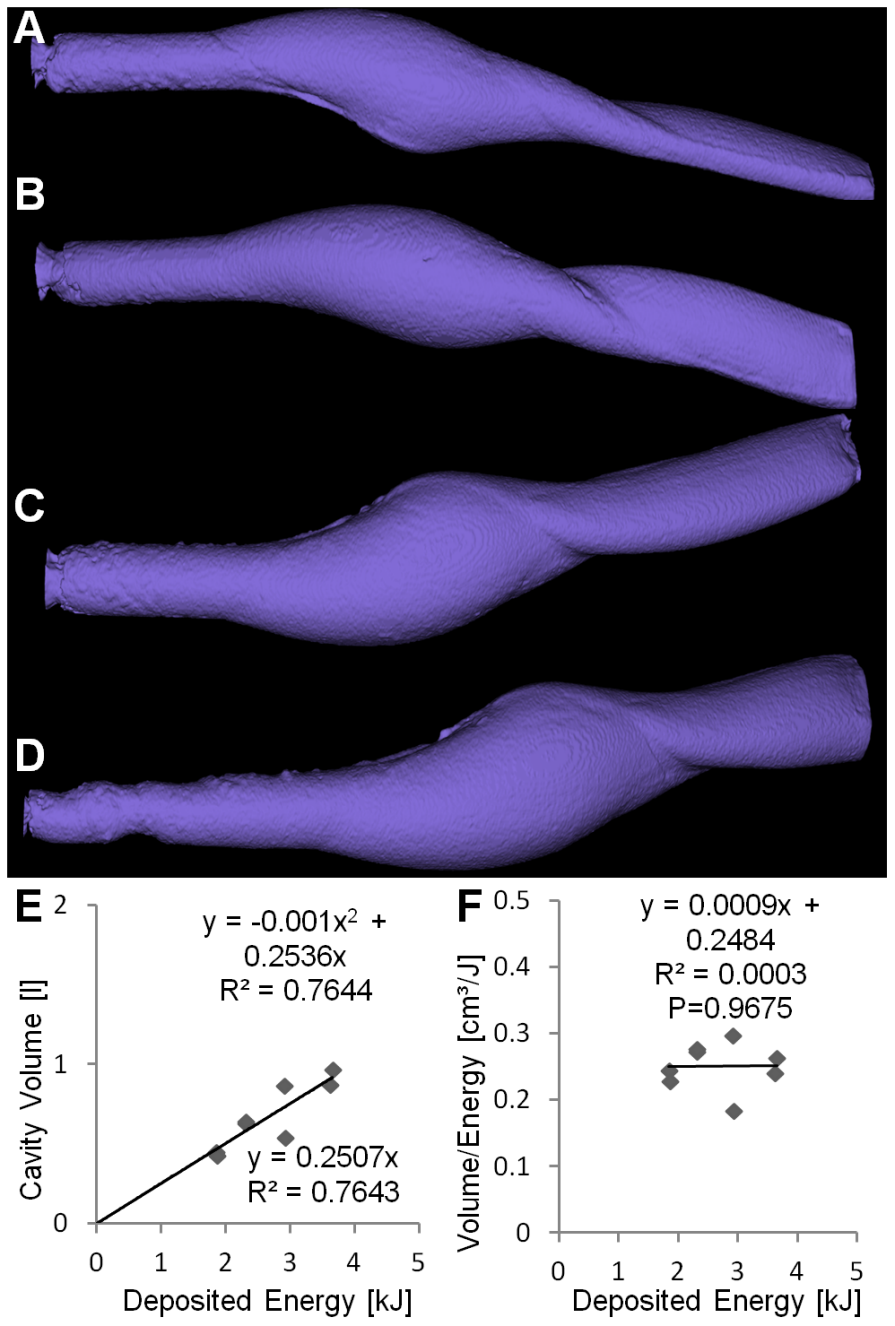
Non-deforming brass bullet (ILS). (A–D) Cavities at increasing energies. The tumbling behavior of the ILS can be seen from the cavity shape. (E) Cavity volume plotted over deposited energy with linear and quadratic regression curves showing similar 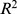. (F) Ratio of volume and deposited energy is constant over the deposited energy.

**Figure 4:**
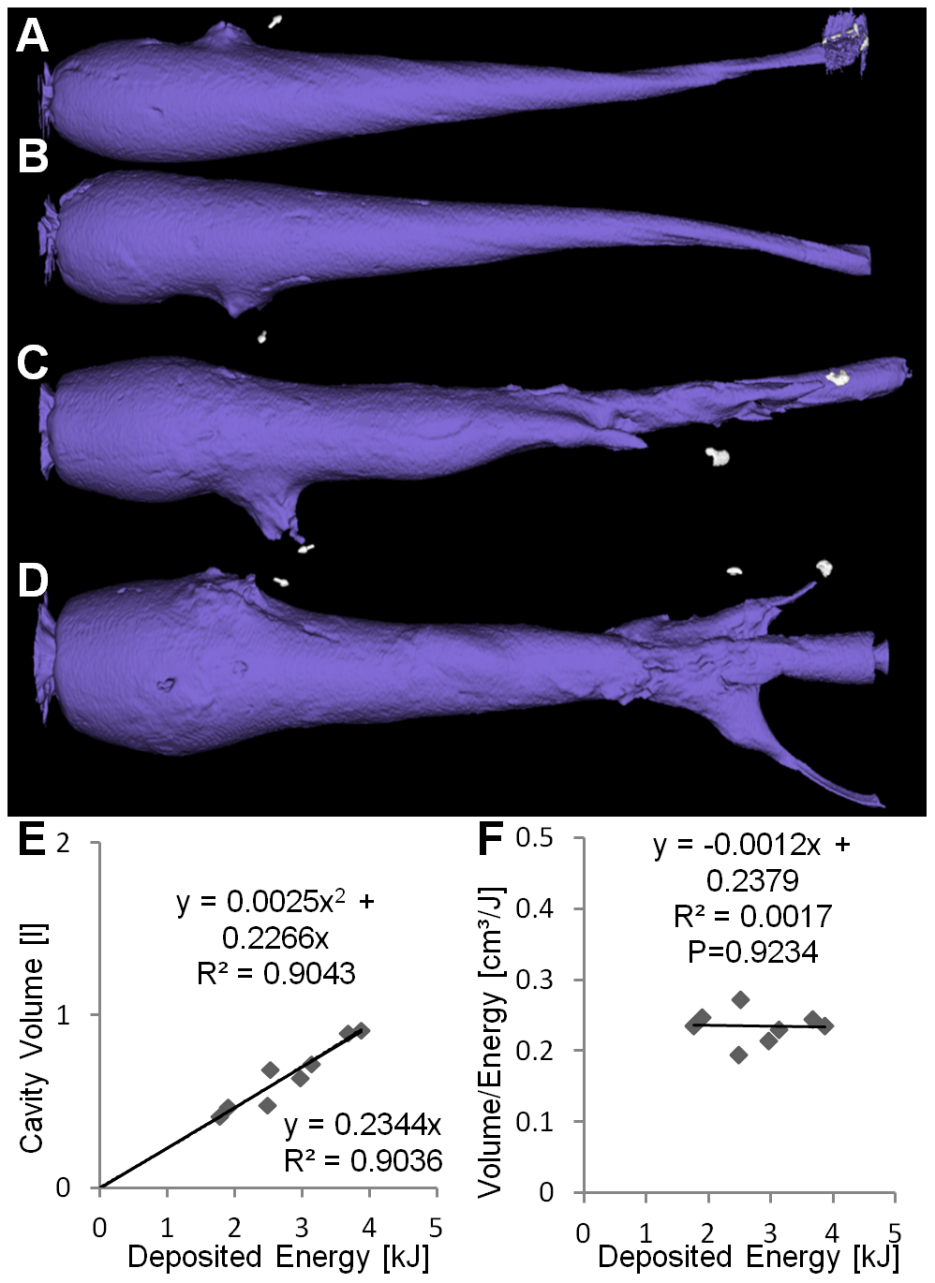
Partially fragmenting copper bullet (TAG). (A–D) Cavities at increasing energies. The aluminum tip which supports the bullet expansion can be seen at similar depths. (E) Cavity volume plotted over deposited energy with linear and quadratic regression curves showing similar 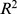. (F) Ratio of volume and deposited energy is constant over the deposited energy.

**Figure 5:**
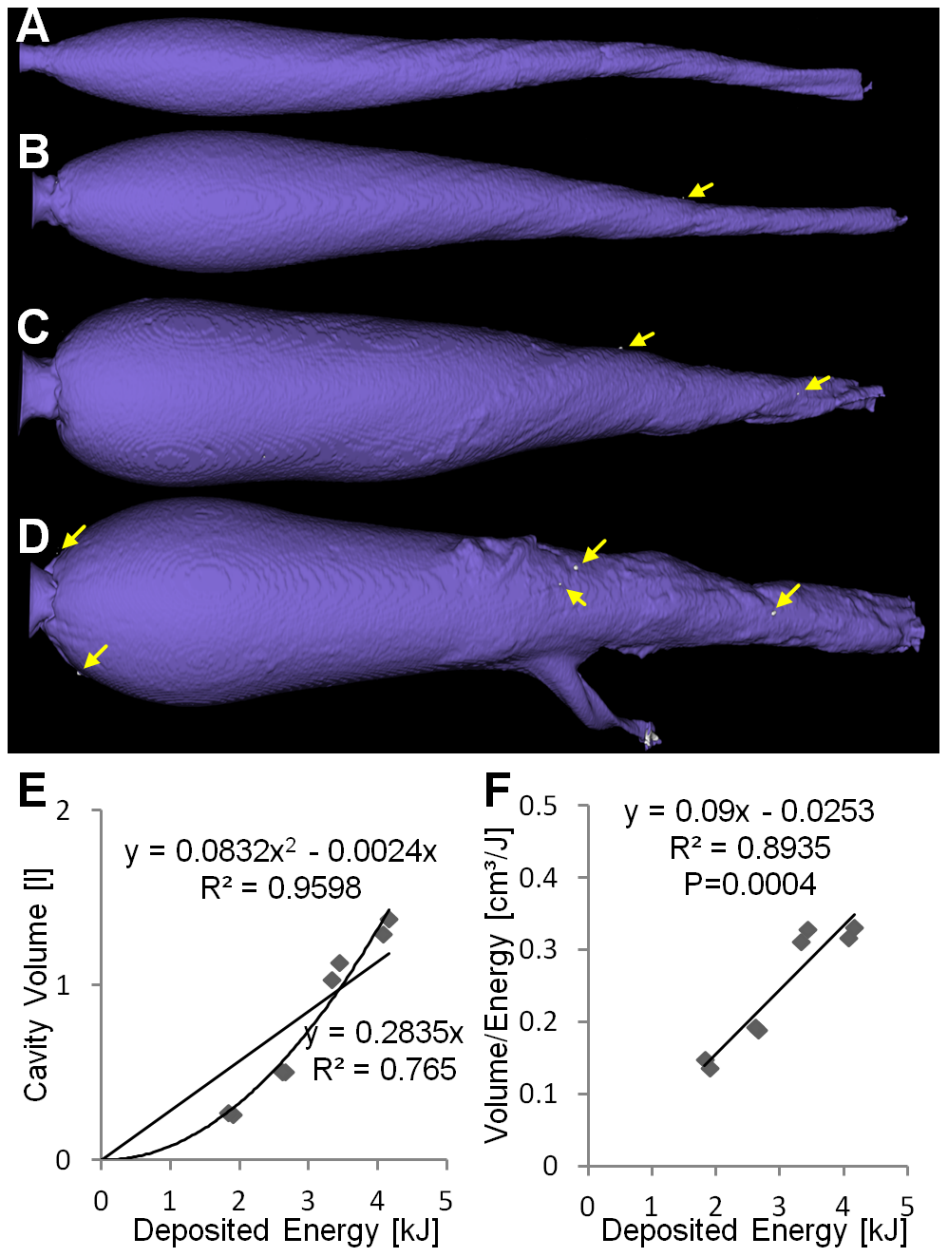
Deforming copper bullet (TSX). (A–D) Cavities at increasing energies. (E) Cavity volume plotted over deposited energy with higher 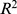 for the quadratic regression than for linear regression. (F) Ratio of volume and deposited energy increases with deposited energy (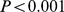). Small fragments are indicated by yellow arrows.

**Figure 6:**
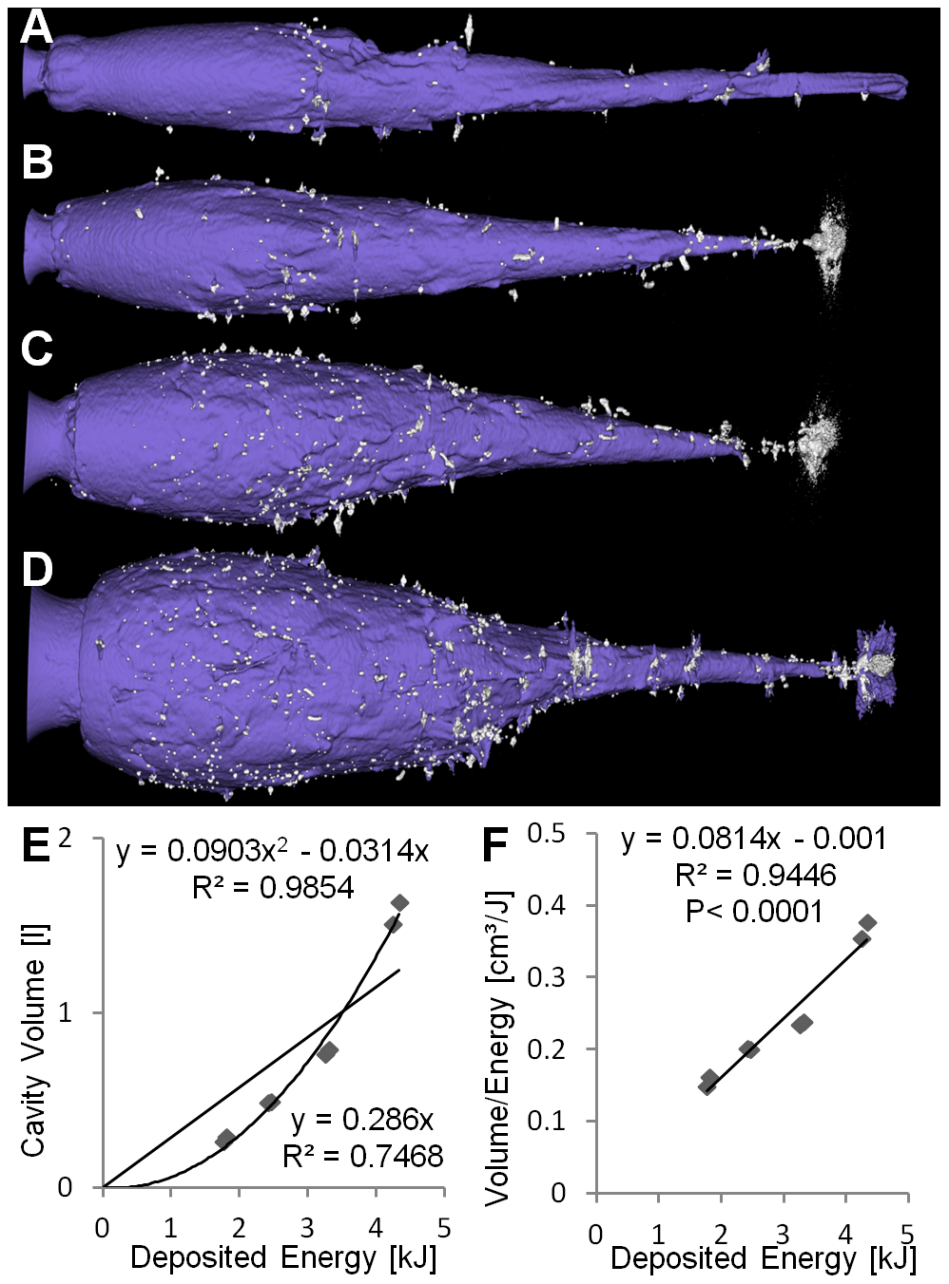
Deforming lead-containing bullet (NVU). (A–D) Cavities at increasing energies. Many metal fragments are visible. (E) Cavity volume plotted over deposited energy with higher 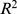 for the quadratic regression than for linear regression. (F) Ratio of volume and deposited energy increases with deposited energy (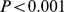).

All bullet types produced cavity volumes which increase significantly with increasing energy deposition 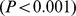. A linear fit seems appropriate for ILS and TAG (Figs. 3, 4) while for TSX and NVU a quadratic fit achieved a much better 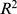 (Figs. 5, 6). The Akaike information criterion supported these observations, i.e. a linear fit achieved a better score for ILS and TAG while a quadratic fit achieved a better score for TSX and NVU. The coefficients of the regression curves were very similar between ILS and TAG and between TSX and NVU.

The ratio of the cavity volume to the deposited energy hardly depends on the deposited energy for ILS and TAG, in contrast to TSX and NVU, where a significant 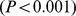 relationship could be found (Figs. 5, 6). Therefore this ratio cannot be assumed to be a soap-dependent constant in these experiments. The slopes of the regression curves were significantly different between bullet types 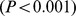. The strong difference between the two copper bullets TAG and TSX is remarkable.

Particularly for low energies, i.e. long shooting distances, ILS and TAG realized higher ratios, i.e. created more cavity volume per energy. If the created volume is a measure of the incurred damage [45], it may put ILS and TAG at an advantage for low energies, i.e. long distances. It should be noted that the energy upon impact is dependent on the shooting distance, the initial speed, and the bullet-dependent ballistic coefficient. In our experiments, the bullets released most of their energy in the block (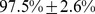).

### Metal Fragments

**Figure 7:**
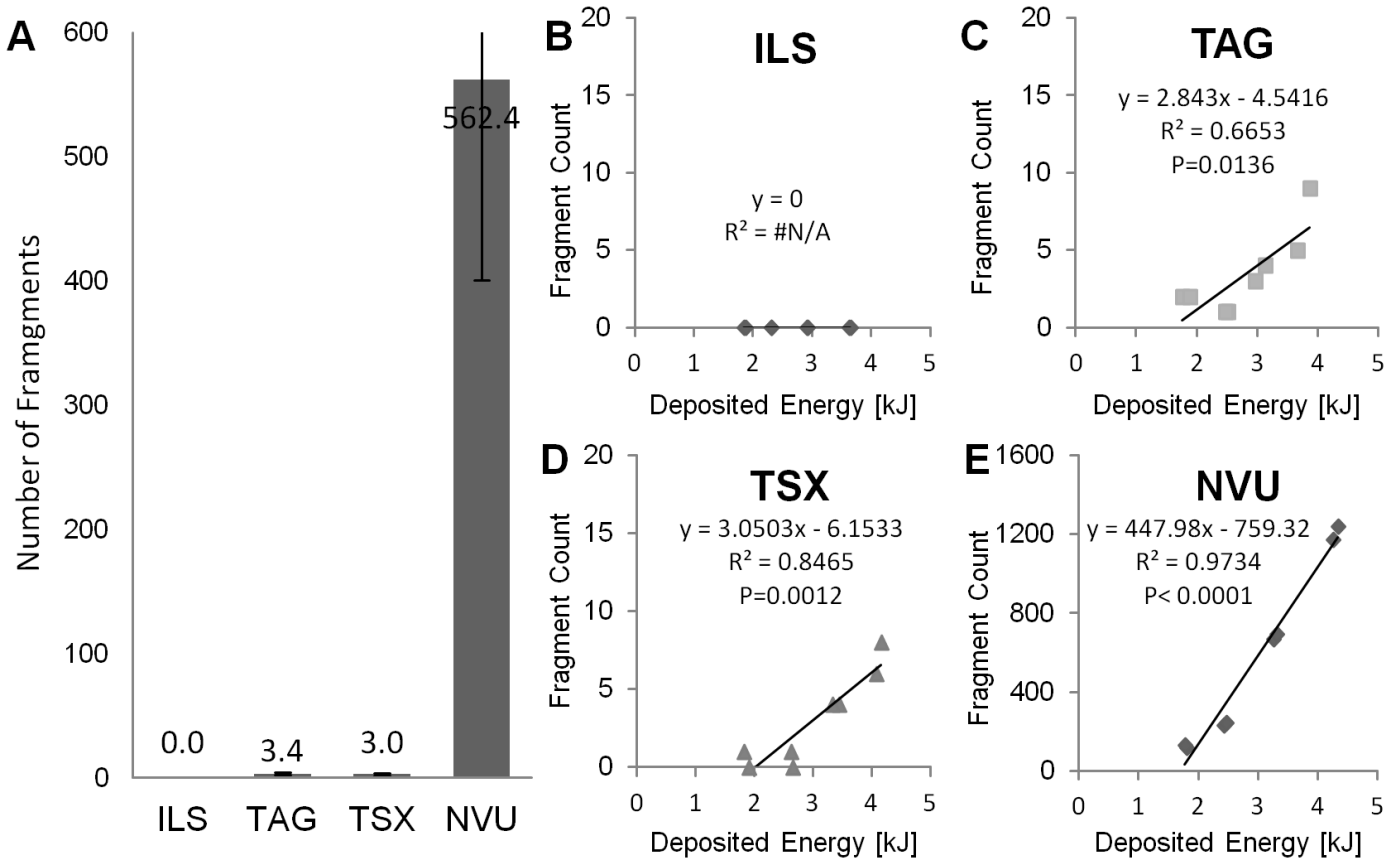
Number of fragments. (A) Significantly more fragments occur for the lead-based bullet compared to all lead-free bullets (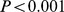). No fragments occur for the brass bullet. (A–E) The number of metal fragments over the deposited energy.

In contrast to the other bullets, absolutely no fragments were found for ILS (Fig. 7). The average number of fragments was 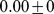 for ILS, 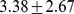 for TAG, 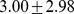 for TSX, and 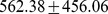 for NVU, and these numbers differ significantly between NVU and other bullets types 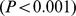. The number of fragments increases significantly with the deposited energy for TAG, TSX, and NVU (Fig. 7). The slopes of the regression lines differ significantly between bullet types 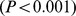. The lead fragments are located closely around the cavity, i.e. do not penetrate deep into the soap, probably due to their small size (Fig. 6). Some copper fragments are penetrating deeper, presumably due to their larger size (Figs. 4, 5).

**Figure 8:**
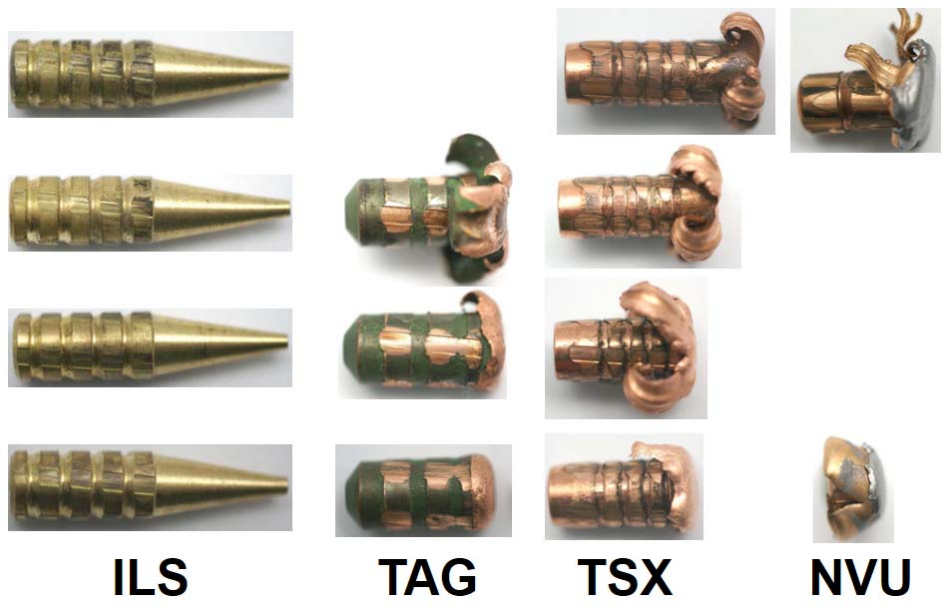
Exiting fragments. The exiting fragments where collected and weighed to calculate the deposited energy. Fragments are shown for increasing speeds from top to bottom. Pictures were only taken when fragments exited the soap block.

The relative weight of the exiting fragments at the highest speeds was 99.4% for ILS, 64.9% for TAG, 73.6% for TSX, and 54.7% for NVU, i.e. highest for the non-deforming ILS bullet and lowest for the lead containing NVU bullet (Fig. 8).

### Cavity Shape

**Figure 9:**
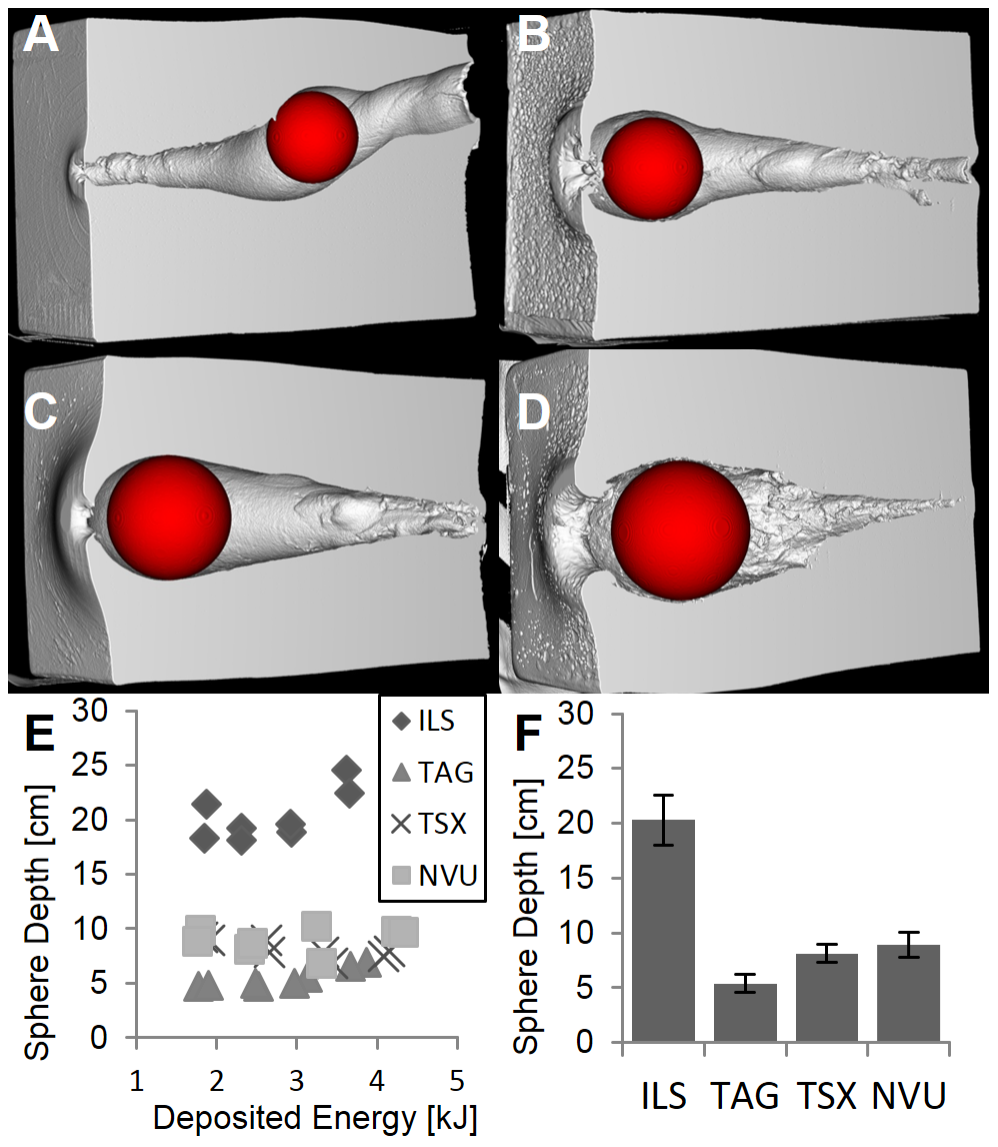
Depth of maximal damage. Position of a maximal fitting sphere was determined for each cavity. (A–D) Examples are shown for ILS, TAG, TSX, and NVU at highest speeds. (E) The depth (along the longest block axis) over deposited energy. (F) Depths differ significantly 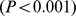 between all bullet types except between TSX and NVU.

The bullets differ in the way they release their energy and therefore in the resulting cavity shape. The non-deforming brass bullet ILS tumbles consistently at around 20 cm depth, thereby increasing its cross-sectional area (Fig. 3). The copper bullet TAG realizes an early opening through an aluminum tip (Fig. 4). The opening of the copper bullet TSX is caused by a drilled hole in the tip and occurs more continuously which results in a tear-shaped cavity similar to that of the lead-containing NVU (Figs. 5, 6). The depth of maximum damage was significantly 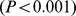 different between all bullet types, except between TSX and NVU (Fig. 9). It was highest for ILS (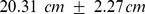), lowest for TAG (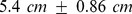), and similar for TSX (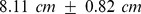) and NVU (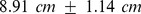).

**Figure 10:**
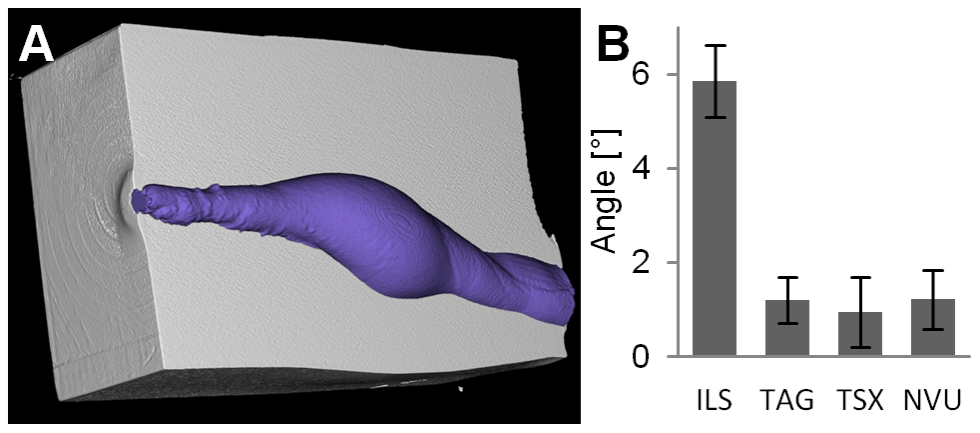
Deviation angle. (A) Virtually cut soap block and cavity show the deviation of the ILS bullet after the tumbling phase. (B) The deviation angle is significantly higher 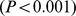 for ILS compared to all other bullet types.

The angles between cavity and soap block (Fig. 10) were 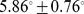 for ILS, 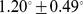 for TAG, 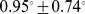 for TSX, and 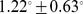 for NVU and differed significantly between ILS and the other bullet types only 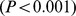.

### Cutting vs. CT-Method

**Figure 11:**
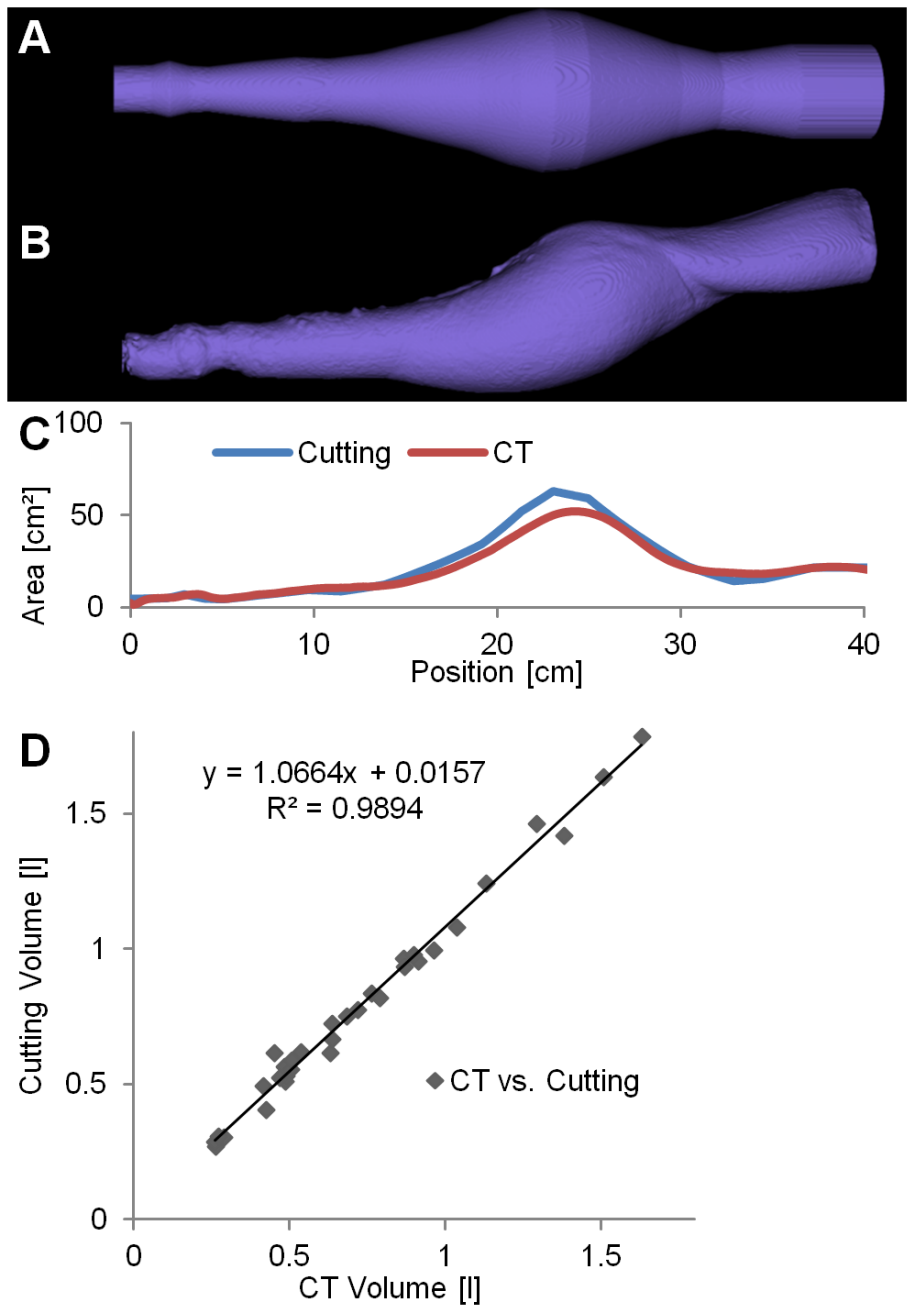
Cutting vs. CT-Method. (A) The cutting method results in piecewise truncated cones which cannot accurately represent elliptical cross-sections. (B) Using the CT images, an accurate model of the cavity is created. (C) For both methods, the cross-sectional area is plotted over the depth. (D) The cavity volumes of 32 blocks measured by both methods show a strong correlation.

The correlation between volumes measured by CT and cutting was very strong 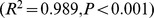 (Fig. 11), however, the cavity volumes measured by CT (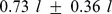) were significantly (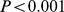) lower than the volumes measured by the cutting method (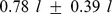), indicating a systematic error (Fig. 11). The relative differences of cutting vs. CT were 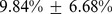. Both methods can be used to estimate the efficacy as function of the penetration depth by computing the cross-sectional area (Fig. 11C). Particularly in regions with elliptical cross sections the cutting method may lead to incorrect results since it assumes circular cross-sections.

**Figure 12:**
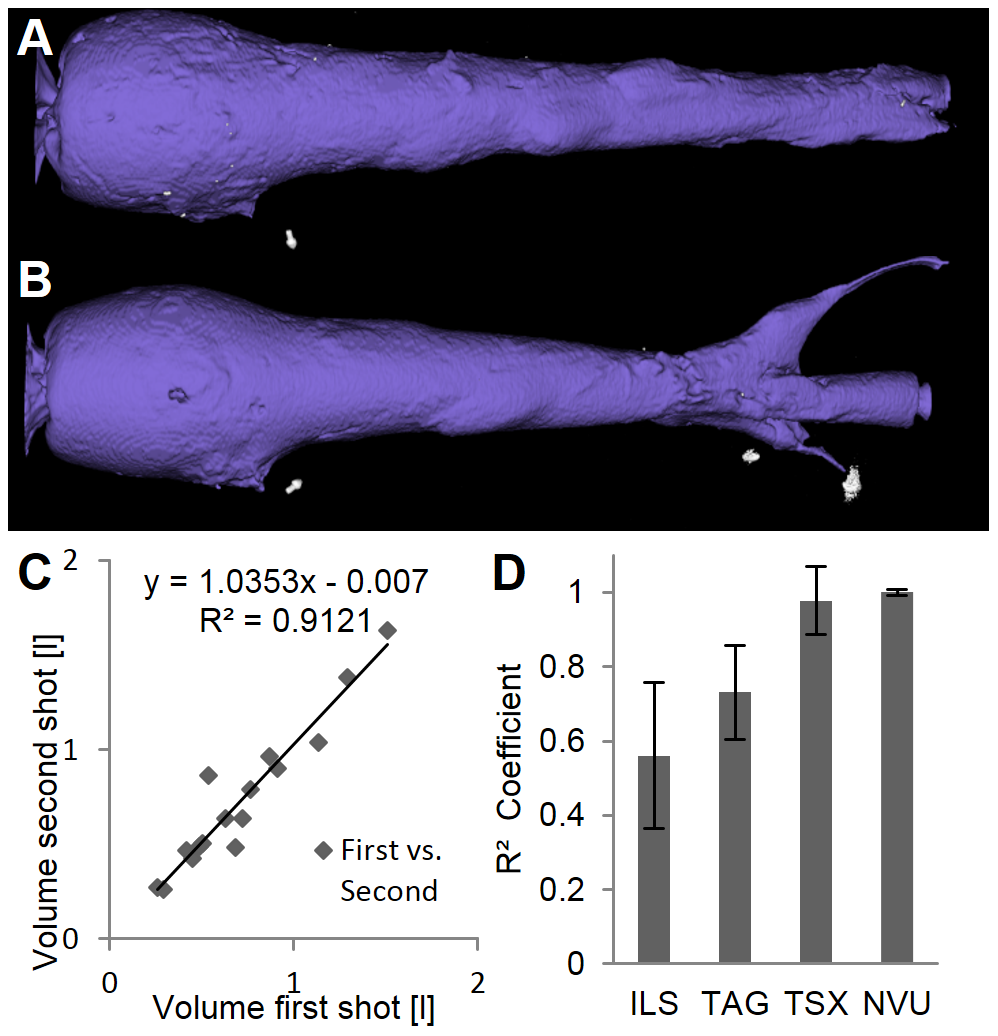
Reproducibility. Reproducibility was assessed by repeating shots. (A, B) Two cavities for the TAG bullet at 900 m/s appear similar except for small parts created by unpredictable fragments. (C) A strong correlation of the volumes between all first and second shots is found (n = 16), showing high reproducibility. (D) The correlation, i.e. reproducibility is highest for NVU and TSX.

The reproducibility of the volume measurements was assessed by repeating each shot under laboratory identical conditions. When correlating the CT-based volumes of all first and second shots, a correlation of 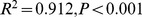 was found (Fig. 12), which was slightly higher than for the cutting method (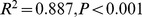), which can be explained by an increased accuracy of the CT-method.

## Discussion

We investigated the terminal ballistic properties of different bullet types in ballistic soap as surrogate to animal tissue. To comprehensively characterize a bullet, we performed shots at 4 different speeds, which we measured using light barriers. Our selection of the three lead-free bullets was meant to cover dimensionally stable, partially fragmenting and deforming lead-free bullet types, and the selection of a commonly used lead-based bullet was based on a previous study [37]. We did not consider the ballistic coefficient of the bullets, the type and amount of powder, and the barrel length. All these affect the relationship between shooting distance and bullet speed upon impact. We neglected these aspects because of the high variety of possible combinations and because these combinations essentially reduce to a certain bullet having a certain speed upon impact and well-trained hunters are capable of estimating these numbers for their rifles.

Our analysis of the cavity volume shows that the relationship between deposited energy and cavity volume cannot be described by a simple linear function in general, as has been shown for hand guns and low energies [45]. While for ILS and TAG, a linear fit adequately describes the relationship (Figs. 3, 4), for TSX and NVU a quadratic fit is more appropriate (Figs. 5, 6). There are two explanations for the differences between bullet types tested here. ILS and TAG require no or little energy for deformation because ILS does not deform at all and the deformation of TAG is supported by an aluminum tip. Therefore, less energy is lost for deformation which may be particularly relevant for low energies where ILS and TAG excel at their energy-to-volume conversion. The other reason could lie in the position and shape of the cavity. Since soap is generally incompressible, the soap masses have to get displaced towards the outside of the block, which is visible on the outer surfaces of the block (Fig. 1). Therefore, an initial amount of energy is consumed to accelerate the soap masses which may result an improved conversion rate at higher energies. The delayed expansion of TSX and NVU which results in an energy deposition deeper in the soap block may result in a decreased volume-to-energy ratio at low energies and an increased ratio at high energies. It was reasoned that the cavity volume is a measure for the stretching and tearing of tissue and therefore for the incurred wound damage [45]. Since we show that the cavity volume cannot be predicted from the deposited energy in general, the cavity volume should be measured directly to assess the damaging potential instead of estimating it from the deposited energy. We will include this insight into an ongoing study [39] where we associate field reports with corresponding soap blocks to assess which features of the cavities correlate with observed effects, such as the flight distance.

The three-dimensional CT acquisition allows visualization of metal fragments and an assessment of their number and position. The lead-containing bullet creates hundreds of small lead fragments (Fig. 7) which remain near the cavity. In this homogeneous soap, the lead-free bullets create much less but sometimes larger fragments which tend to intrude deeper into the tissue surrogate. For the TSX bullet, we found more fragments than expected from a previous study [10]. In that study the Barnes XLC, the predecessor of the Barnes TSX, was used. Furthermore, 2D radiography was used which may have limited sensitivity with respect to small fragments, especially in heterogeneous tissue. We may not be able to detect very small metal fragments or separate closely positioned ones due to the limited CT resolution. To assess the size distribution of the fragments more accurately, we consider imaging of soap pieces in special µCT devices [54].

Upon impact, a long range hunting bullet needs to expand from its aerodynamic shape into a shape with higher cross-sectional area. The three lead-free bullets rely on different means, i.e. tumbling, expansion based on an aluminum tip, and expansion based on a drilled hole. Consequently, the cavity shape varies between these bullet types resulting in different deviation angles (Fig. 10) and depths of maximum damage (Fig. 9). For hunters it is important that the cavity, i.e. the damage dealt, extends along the line of shooting. Otherwise the shot is not predictable and may result in unnecessary suffering of wounded animals escaping or in collateral damage of other animals in the near range. Still, the angle is around 6° for ILS which should be tolerable for most applications. Full metal jacket bullets, which are rarely used for hunting, are likely to create much higher deviation angles [45].

The high resolution CT allows non-destructive imaging of the soap block and generation of a detailed model of the cavity. In contrast to the cutting method and previous work using CT to image soap blocks [46], we performed a voxel-wise segmentation which is more accurate for cavity regions with non-circular cross-sections. Currently, the segmentation of the cavity is created interactively, i.e. the user has to provide some input. In most cases only the start and end of the cavity has to get provided since the other borders of the cavity are confined by the soap. While this only takes a few minutes it could be eliminated completely if an automated segmentation algorithm would be developed [55]. While a strong correlation appears between the volumes determined by cutting and by CT (Fig. 11), there seems to be a systematic error leading to a consistent overestimation of the cutting-based volume compared to the CT-based volumes. Since CT is highly accurate for volume measurements [56], it is likely that this problem arises during the cutting procedure or the subsequent photographic measurement which involves a perspective transformation which may be difficult to calibrate. We did not assess the volume using water filling because this would have affected subsequent measurement steps by dissolving the soap. Nevertheless, such measurements would allow a better assessment of the accuracy of CT and cutting methods.

In this study we investigated 4 different bullet types at 4 impact speeds, leading to 16 different configurations. Due to differing bullet weights and ballistic coefficients, it is not appropriate to directly compare measurements, e.g. cavity volumes, between bullet types. Instead, we performed parametric curve fitting, e.g. cavity volume as a function of deposited energy, which allows comparison of the parameters between bullet types. While repetitions are not required for the used statistic methods, we repeated all shots under laboratory identical settings to increase the number of measurements. Furthermore, this allowed assessment of the reproducibility of ballistic soap experiments, e.g. by comparing the cavity volumes of first and second shots (Fig. 12). We found that the lead-containing NVU and the lead-free TSX bullets achieved a similarly high reproducibility, while reproducibility was lower for the lead-free ILS and TAG bullets, probably as a consequence of the tumbling behavior and the creation of large fragments, respectively.

The behavior of bullets in ballistic soap is not assumed to be equivalent to the behavior in real inhomogeneous tissue, particularly due to the presence of bones which are often hit when targeting at the heart. Soap has a similar density as muscle, blood and organs such as liver, heart, kidneys, and spleen, while lung tissue is around 60% less dense. Real tissue differs in terms of elasticity, with lung and muscle being considerably less vulnerable to deformation than liver and kidney tissue [41]. Bone fragments and other heterogeneous tissue parts could get embedded into the soap block to generate more realistic structures. This would introduce more variability, however, and more repetitions would be required to robustly characterize the bullets. Furthermore, our results pertain to 7.62 mm ammunition, and extrapolation to other calibers, especially those with lower speeds or different bullet weights, may be inappropriate.

Our study shows that considerable differences exist between lead-free bullets with respect to the energy-to-volume conversion, the number of fragments, and the cavity shape. Interestingly, the lead-free TSX bullet is remarkably similar to the lead-containing NVU bullet in all parameters which we quantified, except for the number of fragments.

While our study mainly addresses questions regarding lead-free bullet development and selection for hunting, the knowledge of the behavior of different bullet types may also be useful for forensic investigations [57]. Furthermore, the 3D rendering of the cavity may be useful to understand terminal ballistic effects and for educational purposes. Therefore, our methods should be useful to support hunters, wildlife managers, manufacturers, policy-makers and scientists in the ongoing transition towards lead-free hunting ammunition.
